# 

**Published:** 1846-04

**Authors:** 


### WESTERN JOURNAL

OF

### MEDICINE AND SURGERY.

### EDITORS.

---

DANIEL DRAKE, M.D.,

PROFESSOR OF PATHOLOGY AND THE PRACTICE OF MEDICINE IN THE MEDICAL INSTITUTE OF LOUISVILLE:

LUNSFORD P. YANDELL, M.D.,

PROFESSOR OF CHEMISTRY AND PHARMACY IN THE SAME:

THOMAS W. COLESCOTT, M.D.

### TO CORRESPONDENTS.

We have received communications from Drs. Gordon, Fee, and Boling which will appear in our next number.

---

### BOOKS RECEIVED.

We are indebted to the publishers for the second volume of Velpeau's great work on Surgery. An extended review of it will be prepared for an early number. We have also received a neat volume entitled a "Compendium of Chapman's Practice," which we shall sieze the first leisure hour to notice under the proper head.

The Western Journal of Medicine and Surgery is published monthly by the undersigned, at the corner of Main and Fifth streets, Louisville, at $5 per annum, payable in advance. Each number contains 96 pages, making two volumes in the year of more than 550 pages.

Contributions to its pages by the Physicians of the Valley of the Mississippi, addressed to the Editors, are respectfully solicited.

Letters on business, to be addressed, postage paid, to the publishers.

*January*, 1844. PRENTICE & WEISSINGER.

### PROSPECTUS OF THE SECOND SERIES

OF THE

### *Receipts of Medical Journal for March*, 1846.

| | |
|---|---|
| Dr. W. G. Gordon, Oak Grove, Mo., | $5 00 |
| Dr. J. W. Long, Paris, Mo., | 5 00 |
| D. J. H. Miner, Bedford, Ky., | 5 00 |
| Dr. P. Winfree, New River, La. | 5 00 |
| Dr. J. C. Harris, Wetumpka, Ala., | 10 00 |
| Dr. B. F. Summers, County, | 10 00 |
| Dr. A. M. Ayres, Salem, Miss., | 5 00 |
| Dr. J. Greer, Independence, Mo., | 2 50 |
| Dr. H. Strong, Cold Spring. Miss. | 5 00 |
| Dr. W. F. Brown, Spring Hill, Tenn., | 5 00 |
| Dr. B. Ragan, Numa, Ind., | 5 00 |
| Dr. V. T. West, Union, Ind., | 5 00 |
| Dr. S. Loring, Paris, Tenn.. | 1 25 |
| Dr. B. B. Thornton, Springfield, Mo. | 5 00 |
| Dr. J. B. Nash, Dixon, Ill., | 10 00 |
| Dr. J. S. Crawford, Vicksburg, Miss., | 5 00 |
| Dr. T. H. Todd, Memphis, Tenn., | 5 00 |
| Dr. W. D. Harris, Utica, Miss., | 5 00 |
| Dr. A. Moore, Memphis, Tenn., | 5 00 |
| Dr. J. W. Gray, Hernando., Miss., | 10 00 |
| Dr. A. J. Ellis, Bellmont, Miss., | 5 00 |
| Dr. J. L. Riddell, New Orleans, La., | 5 00 |
| Dr. T. D. Isom, Oxford, Miss., | 5 00 |
| Dr. B. Nailor, Vicksburg, Miss., | 30 00 |
| Dr. C. H. Spillman, Keene, Ky. | 5 00 |
| Dr. M. Pendleton, Stanford, Ky., | 10 00 |

